# Supplementary material for: Allosteric coupling of sub-millisecond clamshell motions in ionotropic glutamate receptor ligand-binding domains
Source: Commun Biol. 2021 Sep 9;4:1056. doi: 10.1038/s42003-021-02605-0 (PMC8429746; doi:10.1038/s42003-021-02605-0)
Supplement: Supplementary file 2 — Supplementary Information [file 42003_2021_2605_MOESM2_ESM.pdf]

Supplementary Information

Allosteric coupling of sub-millisecond clamshell motions in ionotropic glutamate receptor ligand-binding domains

Suhaila Rajab[1], Leah Bismin[1], Simone Schwarze[1], Alexandra Pinggera[2], Ingo H. Greger[2] & Hannes Neuweiler[1,*]

[1]Department of Biotechnology & Biophysics, Julius-Maximilians-University Würzburg, Am Hubland, 97074 Würzburg, Germany.

[2]Neurobiology Division, Medical Research Council Laboratory of Molecular Biology, Francis Crick Avenue, Cambridge CB2 0QH, United Kingdom

*Correspondence to: hannes.neuweiler@uni-wuerzburg.de

Supplementary Figures

Supplementary Figure 1

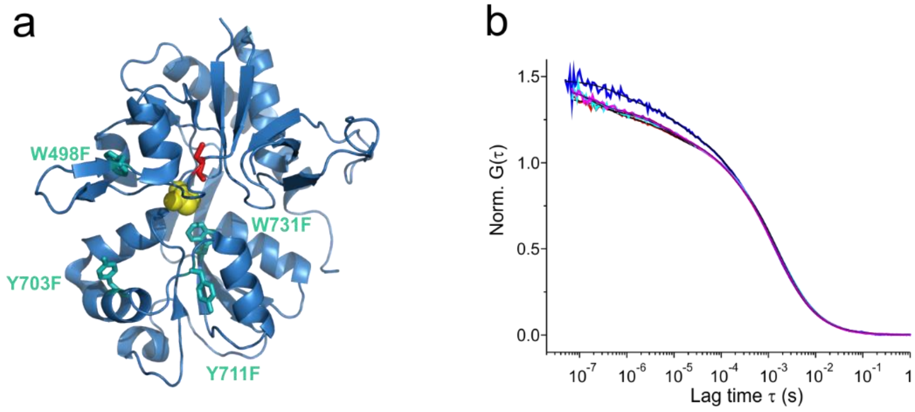

Supplementary Figure 1: **Native Trp and Tyr side chains in GluN1 LBD do not quench fluorescence of the label AttoOxa11**. (**a**) Structure of GluN1 LBD (PDB ID 1PB7) in cartoon representation. The engineered side chain A480C used as label position for AttoOxa11 is highlighted as red sticks. Native Trp and Tyr side chains in the vicinity targeted by mutagenesis are shown in cyan stick representation (mutations W498F, W731F, Y703F, and Y711F). (**b**) ACFs, ($G(\tau)$), recorded from fluorescently modified apo GluN1 LBD mutants W498F (red), W731F (blue), Y703F (cyan), and Y711F (magenta). Black lines are fits to the data using a model for molecular diffusion containing a sum of three single-exponential decays. ACFs are normalized to the average number of molecules in the detection focus for reasons of clarity.

Supplementary Figure 2

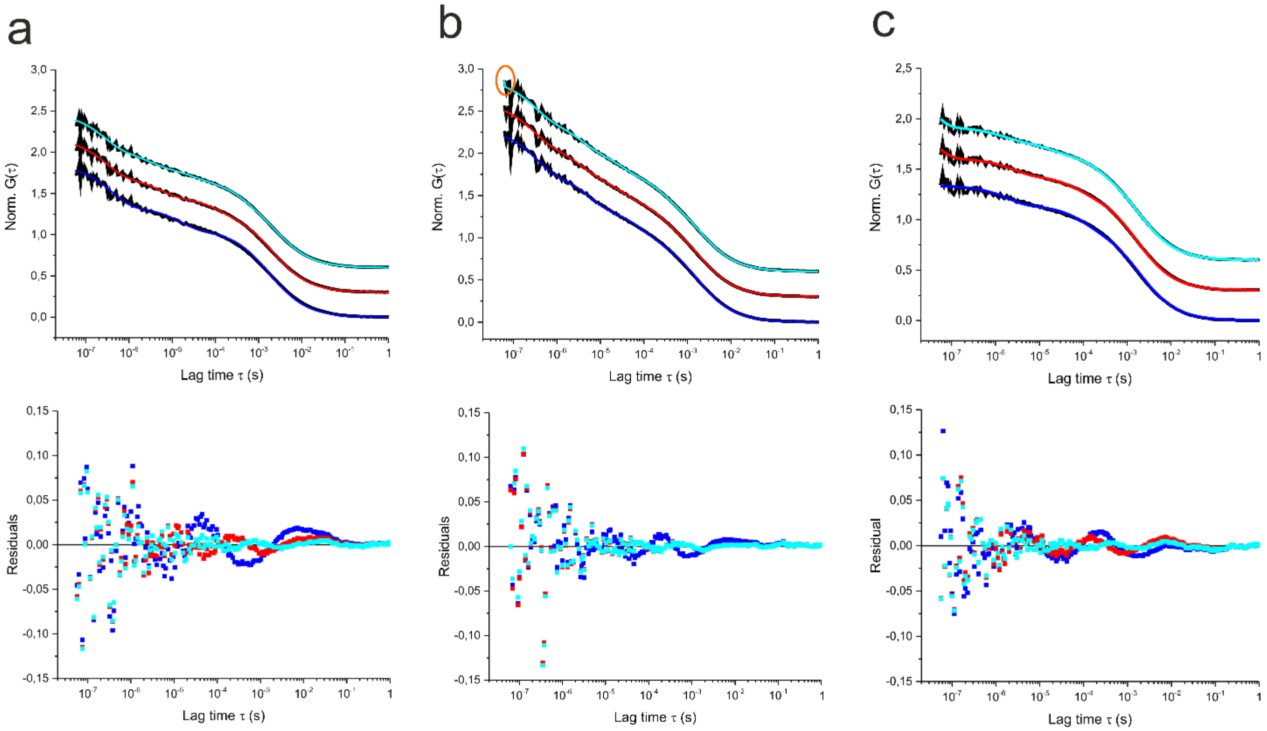

Supplementary Figure 2: **Quality of fits to the ACFs of LBDs applying models using varying numbers of exponentials**. (**a-c**) ACFs, (G(τ)), normalized to the average number of molecules in the detection focus, recorded from the constructs GluA2-LBD-G446C-T685W (a), GluK1-LBD-K503C-K734W (b), and GluN1-A480C (c). Fits to the data applying models containing a sum of three, four, and three exponential decays, respectively, as described in the main text, are shown in red. Fits to the data using the same model but removing one exponential or adding one exponential are shown in blue and cyan, respectively. The corresponding residual plots are shown in the lower panels. A model containing five exponentials applied to the data of GluK1-LBD-K503C-K734W yields a physically unreasonable decay detected on the first two data points, indicated by the orange circle in panel (b).

Supplementary Figure 3

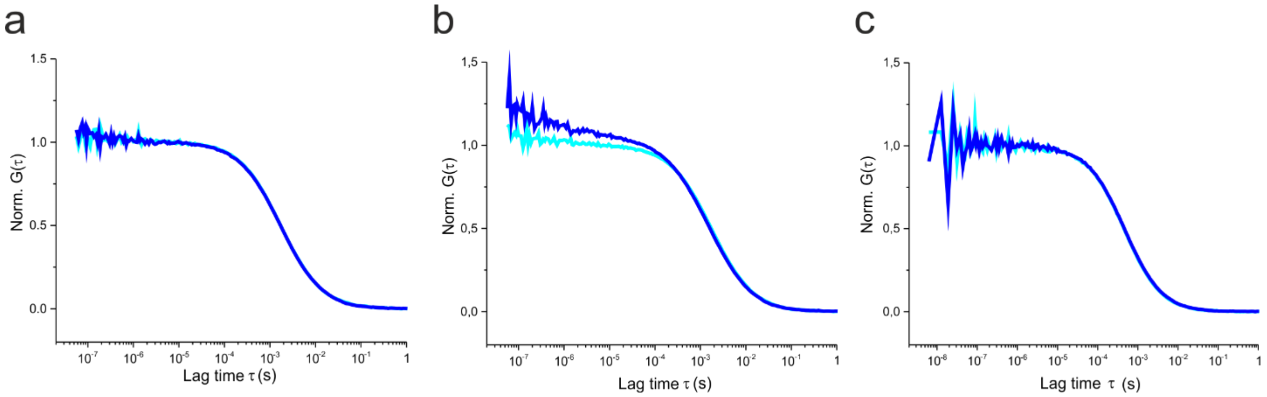

Supplementary Figure 3: **Test of the influence of ligands on the label AttoOxa11**. (**a**) ACFs, (G(τ)), recorded from GluA2-LBD-G446C in absence (blue) and presence (cyan) of 1 mM L-Glu. (**b**) ACFs recorded from GluK1-LBD-K503C in absence (blue) and presence (cyan) of 1 mM L-Glu. (**c**) ACFs recorded from AttoOxa11 in absence (blue) and presence (cyan) of 1 mM Gly. ACFs are normalized to the average number of molecules in the detection focus for reasons of clarity.

Supplementary Figure 4

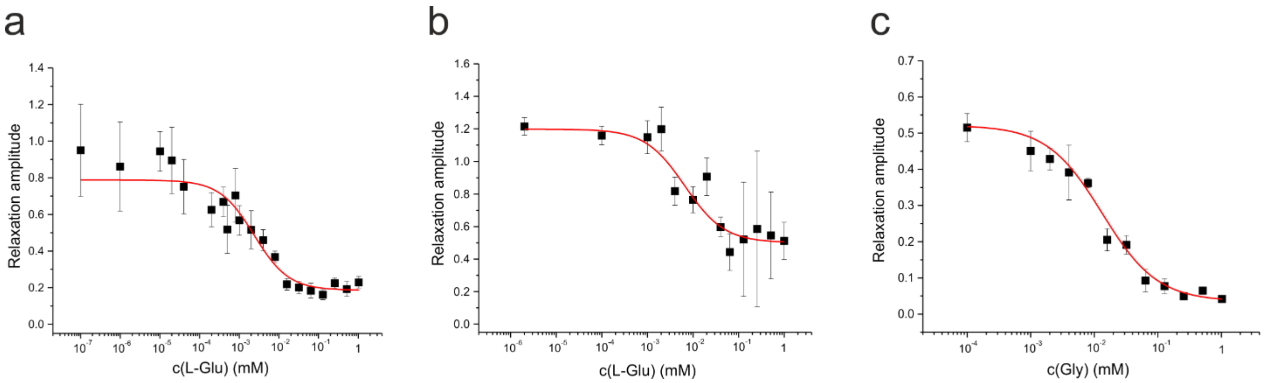

Supplementary Figure 4: **Ligand-binding isotherms measured from fluorescently modified LBDs using PET-FCS**. (**a**) Sum of relaxation amplitudes that decay upon binding of ligand, measured from GluA2-LBD-G446C-T685W, plotted versus concentration of agonist. (**b**) Sum of relaxation amplitudes that decay upon binding of ligand, measured from GluK1-LBD-K503C-K734W, plotted versus concentration of agonist. (**c**) Sum of relaxation amplitudes that decay upon binding of ligand, measured from GluN1-A480C, plotted versus concentration of agonist. Red lines

are fits to the data using a model for a protein-ligand binding isotherm. Error bars are propagated s.e. from fits to PET-FCS data.

Supplementary Figure 5

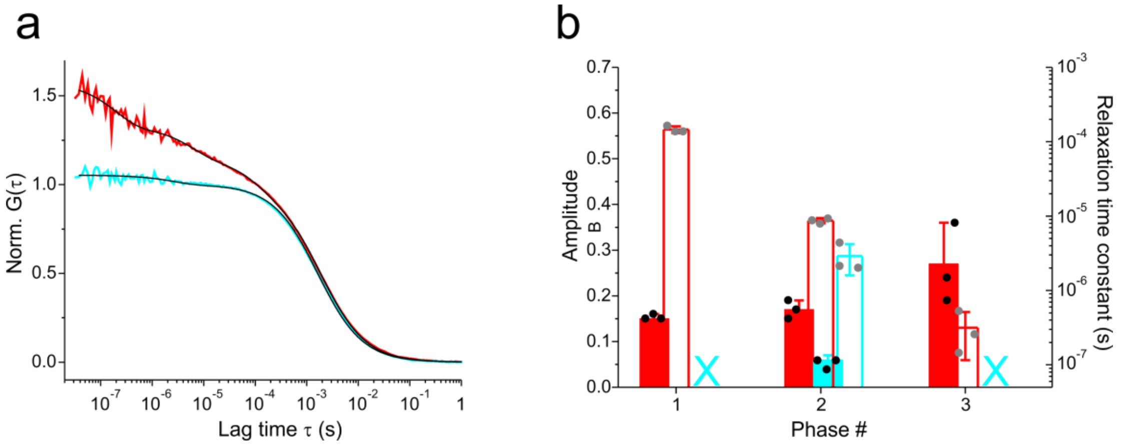

Supplementary Figure 5: **FCS of monomeric GluN1 LBD mutant Y535S**. (**a**) ACFs, (G(τ)), normalized to the average number of molecules in the detection focus, recorded from monomeric GluN1-LBD-A480C-N521Y-Y535S (red), and in presence of excess Gly (cyan). Black lines are fits to the data using a model for molecular diffusion containing a sum of either three or one single-exponential relaxations (indicated in (b)). (**b**) Amplitudes (closed bars) and corresponding time constants (open bars) obtained from exponential fits to relaxations detected in ACFs shown in panel (a). Data sets and color code correspond to data shown in panel (a). A cross (X) denotes a missing (not detected) exponential phase. Error bars are s.d. of three measurements (n = 3).

Supplementary Tables

Supplementary Table 1: Degrees of labelling (DOL) determined from labelling trials of wild-type LBDs and constructs containing engineered Cys residues.

Construct	DOL	DOL (wild-type)
GluA2-LBD-G446C	0.61	0.11
GluK1-LBD-K503C	0.55	0.10
GluN1-A480C	0.59	0.08

Supplementary Table 2: Kinetic parameters derived from fits to ACFs recorded from Trp/Tyr point mutants of fluorescently modified GluN1-LBD-A480C.

	τ_D (ms)	a_1	τ_1 (μs)	a_2	τ_2 (μs)	a_3	τ_3 (μs)
W498F	1.47±0.01	0.13±0.01	117±12	0.14±0.01	7.2±1.8	0.11±0.01	0.38±0.18
W731F	1.41±0.05	0.20±0.02	137±25	0.17±0.01	10±2	0.11±0.01	0.68±0.13
Y703F	1.55±0.08	0.16±0.01	108±23	0.15±0.01	7.4±1.5	0.18±0.08	0.26±0.15
Y711F	1.43±0.05	0.16±0.01	101±12	0.15±0.03	6.7±0.3	0.13±0.03	0.23±0.02

Values are the mean of three measurements (n = 3±s.d.)
