# Supplementary material for: Allosteric coupling of sub-millisecond clamshell motions in ionotropic glutamate receptor ligand-binding domains
Source: Commun Biol. 2021 Sep 9;4:1056. doi: 10.1038/s42003-021-02605-0 (PMC8429746; doi:10.1038/s42003-021-02605-0)
Supplement: Supplementary file 3 — Description of Supplementary Files [file 42003_2021_2605_MOESM3_ESM.pdf]

Description of Additional Supplementary Files

File name: Supplementary Data 1

Description: Source data underlying graphs and charts.
